# The importance of quantitative trait differentiation in restoration: landscape heterogeneity and functional traits inform seed transfer guidelines

**DOI:** 10.1093/aobpla/plaa009

**Published:** 2020-03-07

**Authors:** Zebadiah G Yoko, Kate L Volk, Ned A Dochtermann, Jill A Hamilton

**Affiliations:** Department of Biological Sciences, North Dakota State University, Fargo, ND, USA

**Keywords:** Alvar, common garden experiment, ecophysiology, genetic variation, Geum triflorum, local adaptation

## Abstract

For widely distributed species, understanding the scale over which genetic variation correlates to landscape structure and composition is critical. Particularly within the context of restoration, the evolution of genetic differences may impact success if seeds are maladapted to the restoration environment. In this study, we used *Geum triflorum* to quantify the scale over which genetic differences for quantitative traits important to adaptation have evolved, comparing the proportion of variance attributed to broad regional- and local population-level effects. *Geum triflorum* is a widely distributed species spanning a range of environments, including alvar and prairie habitats, which have extreme regional differences in soil-moisture availability. Alvar habitats are regions of thin soil over limestone that experience substantial seasonal variation in water availability, from flooding to desiccation annually. This contrasts with prairie habitats, whose deeper soils mitigate irregular flood–desiccation cycles. Using a common garden experiment, we evaluated 15 traits broadly grouped into three trait classes: resource allocation, stomatal characteristics, and leaf morphological traits for individuals sourced from prairie and alvar environments. We quantified the proportion of trait variance explained by regional- and population-scale effects and compared the proportion of regional- and population-trait variances explained across trait classes. Significant regional differentiation was observed for the majority of quantitative traits; however, population-scale effects were equal or greater than regional effects, suggesting that important genetic differences may have evolved across the finer population scale. Stomatal and resource allocation trait classes exhibited substantial regional differentiation relative to morphological traits, which may indicate increased strength of selection for stomatal and resource allocation traits relative to morphological traits. These patterns point towards the value in considering the scale over which genetic differences may have evolved for widely distributed species and identify different functional trait classes that may be valuable in establishing seed transfer guidelines.

## Teaser

Restoration aims to source appropriate seed for success in the restoration environment. However, genetic differences in traits important to plant function may have evolved influencing success of different seed sources when planted in different environments. In this study, we established a common garden experiment using the widespread perennial, *Geum triflorum*, to examine the proportion of trait differences attributable to regional or population environments. Environmental differences associated with regional climate differences significantly influenced the distribution of functional trait variation across seed sources, pointing towards the importance of understanding climate adaptation when recommending seed transfer for restoration.

## Introduction

Understanding the scale over which phenotypic traits evolve is crucial to successful habitat restoration. Both macro- and microevolutionary processes contribute to the evolution of broad- and fine-scale genetic variation for traits important to adaptation (McKay *et al.* 2001; Hamilton *et al.* 2020). However, if quantitative trait differences evolve over varying ecogeographic scales, seed transfer across those scales could impact restoration success. The use of maladapted genotypes can compromise the long-term success of restoration programmes if fitness of translocated individuals is reduced (Langlet 1971; Aitken and Bemmels 2016). This is particularly true for grassland ecosystems, which remain one of the most critically imperilled ecosystems globally due to a combination of anthropogenic conversion and fragmentation (Hoekstra *et al.* 2005; Wimberly *et al.* 2018). Large-scale restoration efforts are needed to restore key ecosystem functioning of these biologically diverse, productive systems (Breed *et al.* 2018; Hamilton *et al.* 2020). However, while extensive research has established seed transfer guidelines in forested ecosystems based primarily on latitude, elevation and climate (Knapp and Dyer 1998; Broadhurst *et al.* 2008; Johnson *et al.* 2010; O’Neill *et al.* 2017), similar guidance is limited in grassland ecosystems. ‘Regional admixture provenancing’ has recently been proposed as a means to increase seed source diversity while preserving local adaptation (Bucharova *et al.* 2019); however, consensus regarding seed transfer guidelines is lacking. Current guidelines are often defined by separate federal, state/provincial, or local jurisdictions, and recommendations may be region- or species-specific (Miller *et al.* 2011; Potter and Hargrove 2012; Crow *et al.* 2018). Consequently, quantifying the ecogeographic scale over which trait differences vary across grassland ecosystems may provide important guidance in establishing seed transfer recommendations.

Identifying the scale and extent over which genetic differences in traits important to adaptation have evolved has been a major goal of restoration ecology (McKay *et al.* 2001; Bucharova *et al.* 2019; Hamilton *et al.* 2020). The evolution of complex traits results from a combination of genetic and environmental variation, as well as their interaction, and can lead to the evolution of locally adapted genotypes (Escudero *et al.* 2003; Wright *et al.* 2004; Martin *et al.* 2007). Locally adapted genotypes exhibit trait values that may be advantageous in their ‘home’ environment, but maladaptive in an ‘away’ environment (Hereford 2009; Johnson *et al.* 2010; Bucharova *et al.* 2017a). Shifts in the direction and magnitude of selection driven by environmental gradients may contribute to the maintenance of geographic variation in phenotypic traits across species’ ranges, contributing to regional- or population-level differences that may impact seed transfer (Manel *et al.* 2003; Messier *et al.* 2010; Hovick *et al.* 2018). However, not all traits are differentiated across region- or population-level scales. This may be due, in part, to a lack of phenotypic variation on which selection may act, or if traits are subject to stabilizing rather than directional selection across environments (Levin 1992; Ackerly 2003). Quantification of the proportion of trait variation attributable to regional- and population-level effects within and across functional trait classes will be valuable in establishing seed transfer recommendations in the future.

For widespread grassland species, identifying and sourcing material for restoration remains challenging, particularly where population trait differences may have evolved in response to varying selective pressures. Understanding the degree to which variance in traits associated with plant function and fitness vary across the landscape will be important to restoration programmes. In this study, we use *Geum triflorum* or Prairie Smoke, to quantify the scale(s) over which trait differences important to plant function have evolved to inform seed transfer in a restoration context.

**Figure 1.**
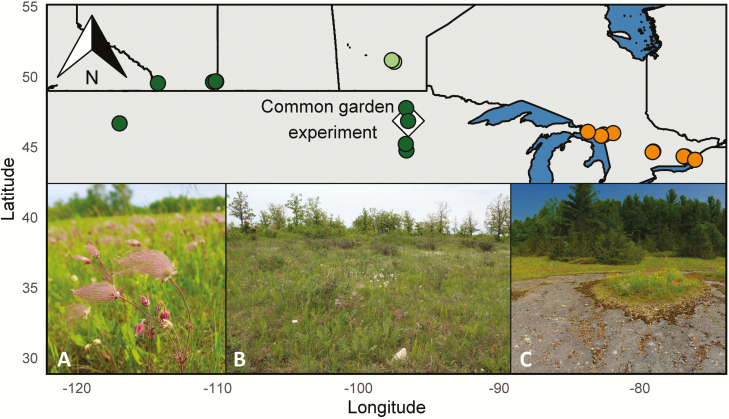
Map and pictures of collection sites of *G. triflorum.* Gold circles represent Great Lake Alvar population collections, two populations represented as light green circles represent Manitoban Alvars and dark green circles represent Prairie populations. Common garden location depicted as a white triangle. Inset pictures represent Prairie (A), Manitoba Alvar (B) and Great Lake Alvar (C) landscapes, respectively.

An herbaceous perennial native to North America, *G. triflorum* has a wide distribution spanning the Great Plains of the USA and Central Canada (hereafter referred to as ‘Prairie’), as well as alvar habitats surrounding the Great Lakes Region and into Manitoba (Fig. 1). As one of the first species flowering in the early spring, *G. triflorum* is a key early-season pollinator resource, and as such plays an important role in ecosystem function. Both prairie and alvar habitats are critically imperilled due to a combination of natural and anthropogenic disturbance (Hoekstra *et al.* 2005; van der Maarel 2006; Catling 2013). Here, we compare regional- and population-trait differentiation for a range of individual quantitative traits and functional trait classes. Prairie and alvar habitats exhibit contrasting environments. Prairie habitats are typically characterized by cold, dry winters and hot, humid summers and experience unpredictable variation in water availability that can be partially mitigated by the presence of thick, nutrient-rich soil (Risser *et al.* 1981; Anderson 2006). In contrast, alvar habitats exhibit shallow soils over dolomitic limestone, and are prone to predictable extremes in seasonal water availability with annual transitions between complete flooding in the spring to total desiccation by early summer (Catling and Brownell 1995; Stark *et al.* 2004). Variation in water availability during the growing season likely contributes to the evolution of genetic differences in quantitative traits between alvar and prairie regions. Here we evaluate 15 quantitative traits organized broadly by functional trait class; including resource allocation, stomatal characteristics, and leaf morphological traits. Trait variation within these classes is often associated with environmental differences (Ackerly *et al.* 2002; Hulshof *et al.* 2013; Martin *et al.* 2017), as all plants balance carbon gain with water loss. To quantify genetic differences in traits for seeds sourced from across the range of *G. triflorum*, we estimate the proportion of trait variances explained by regional and population effects for a number of trait classes. We predict that the scale over which trait classes differ will vary depending on how selection at the regional or population scale impacts the distribution of trait variation across contrasting environments. Identifying the scale over which functional trait classes differ across ecogeographic scales will be informative for developing seed transfer guidelines for restoration for the future.

## Materials and Methods

### Field sampling of *G. triflorum*

In the spring of 2015, seeds from 22 populations of *G. triflorum* were collected across a large portion of the species’ range. Nineteen populations were sampled spanning three distinct ecoregions; including 11 from the Great Lake Alvars (GLA) region, 2 from the Manitoba Alvars (MBA) region and 6 from the Prairie (PRA) region (Fig. 1; Table 1). Forty open-pollinated maternal seed families were collected along a 100-m transect within each population (see detailed sampling methods in Hamilton and Eckert 2007). In addition, two bulk seed collections were provided by Prairie Moon Nursery (SD-PMG, MN-PMG) and one by the United States Department of Agriculture (WA-BLK) from within the prairie region, reflecting seed locally harvested for restoration purpose (Fig. 1; Table 1).

**Table 1.** Source populations of *G. triflorum* collected in 2015 separated by region, along with latitude, longitude and elevation (m) of collection sites. Number of individuals seeded represents the initial number of seeds planted, while the number of individuals in garden represents individuals that germinated and were transplanted to the permanent field site. Percent survival indicates the percentage of individuals persisting in the permanent field common garden from the total initially planted. Distance from garden calculated as greater circle distance between source location and common garden location established at Minnesota State University Regional Science Center.

Populations	Latitude	Longitude	Elevation (m)	No. of individuals seeded	No. of individuals in garden	Percent survival (%)	Distance from garden (km)
Great Lake Alvars							
CAR-NBA	44.68502	−79.05154	268	120	97	81	1368.191
CAR-PSR	44.64526	−79.09458	250	120	75	63	1366.181
MAN-FOX	45.89713	−82.57893	186	120	98	82	1068.226
MAN-KIP	45.87036	−82.53938	183	120	100	83	1071.794
MAN-LCI	45.99426	−81.89436	182	117	77	66	1118.294
MAN-MIS	45.80825	−82.75912	193	119	96	76	1056.428
MI-DRI	46.08578	−83.69201	188	120	103	86	979.8749
NAP-ASS	44.26533	−76.71188	126	120	46	38	1559.351
NAP-CE	44.33003	−76.78968	166	120	70	58	1551.28
NAP-SCH	44.34399	−76.89338	154	120	92	77	1542.945
WNY-CB	44.097639	−76.082861	93	120	92	77	1612.813
Manitoba Alvars							
MB-CRN	51.070942	−97.461336	231	120	91	76	472.9779
MB-MR	51.184289	−97.626839	231	120	87	73	487.3889
Prairie							
AB-HSC	49.636389	−110.33	721	120	5	4	1070.778
AB-LL	49.543611	−114.247222	929	120	108	90	1348.389
AB-RL	49.665278	−110.1075	721	120	0	0	1055.739
AB-RO	49.671944	−110.147222	721	120	6	5	1058.689
MN-PMG	47.7742	−96.6081	267	24	8	33	101.3225
ND-BSP	46.85845	−96.47169	274	120	39	33	1.896
SD-MUD	44.76309	−96.58792	531	120	99	83	234.4168
SD-PMG	45.2186	−96.6336	351	24	11	46	184.0634
WA-BLK	46.685513	−116.971868	786	24	12	5	1558.248
Common garden	46.86913	−96.4522	259	2348	1412	58	

### Common garden experiment

A common garden experiment was established on 7 November 2015 at North Dakota State University. Twenty-two populations were planted across 12 randomized complete blocks. For field-collected populations, 10 maternal seed families were planted per population, including 12 individuals per maternal seed family. For bulk seed collections, 24 seeds were planted including two replicates per block for each source (Table 1). Seeds were treated with a 0.02 % PPM™ fungicide treatment and grown in ‘Cone-tainers™’ (Stuewe & Sons, 158 mL) filled with Sungro horticulture mix soil for ~2 months, following which surviving germinants were re-potted into mini-treepot containers (Stuewe & Sons, 1014 mL). Seedlings were grown for 27 weeks under controlled greenhouse conditions, maintained at a 15 h:9 h daylight to darkness photoperiod with supplemental light from halide lighting at a measured flux density of 0.338 mmol m^2^ s^−1^ and temperatures between 18.3 and 23.9 °C. Plants were watered biweekly, and provided between 10–15 pellets of slow release fertilizer mix (Osmocote 14N-14P-14K) at regular intervals throughout the course of the experiment. In May 2016, surviving seedlings were transferred to a permanent outdoor research facility at the Minnesota State University Moorhead (MSUM) Regional Science Center (Table 1, 46.86913N, −96.4522W). The randomized complete block design was maintained in the field planting. Seedlings were planted directly into soil through cut-outs in a weed barrier to limit competition. Percent survival was calculated following transplant to the outdoor garden as the number of individuals successfully established per population versus the number planted in the initial design (Table 1). The number of individuals established ranged from 5 to 108 per population, with the exception of AB-RL, which exhibited 0 % emergence in the greenhouse (Table 1).

### Measurement of quantitative traits

#### Morphological measurements

**Figure 2.**
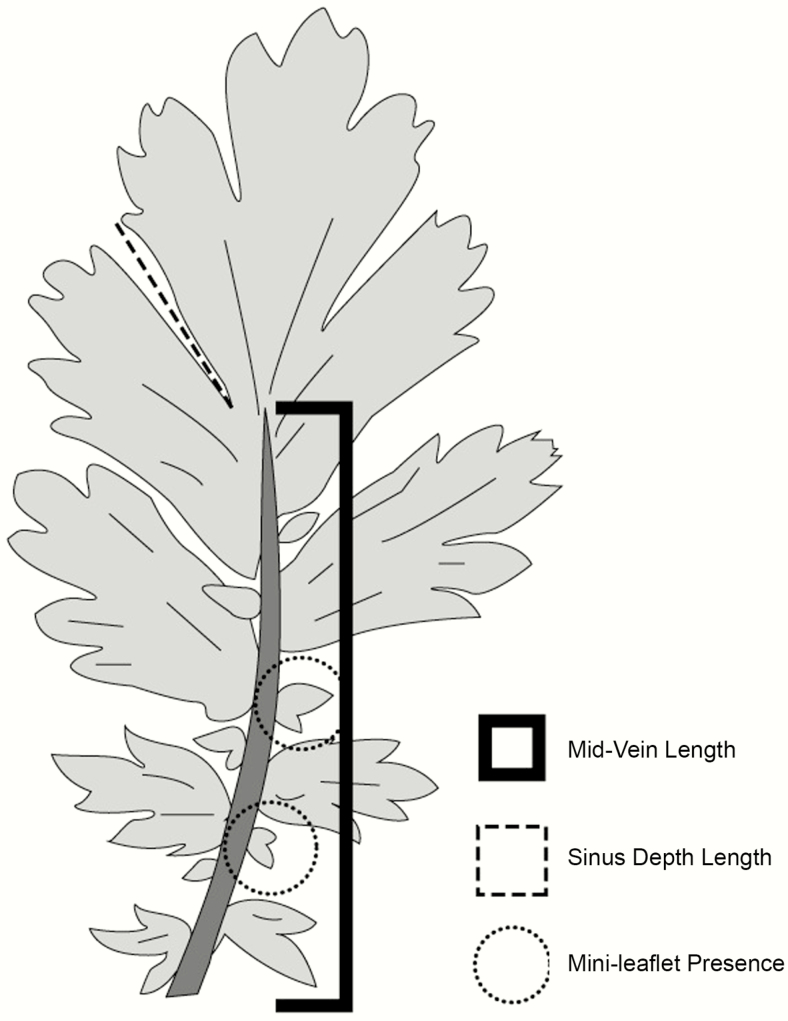
Morphological measurements of leaves of *G. triflorum*. The bracket outlines the length of the midvein, the dashed line represents the length of the major sinus depth, the lightly dashed circle illustrates the mini leaflets, which were assessed as presence/absence, and if present, if leaflets were lobed or not.

Leaf morphological trait variation was quantified for all surviving individuals in the greenhouse prior to transfer to the permanent outdoor research facility. For each individual (*n* = 1396), one leaf was randomly sampled, photographed and measured for variation in midvein length, sinus depth and mini leaflet presence and shape on a 1-cm^2^ grid. Midvein length was measured as the total length of the primary vein per sampled leaf. Sinus depth refers to the depth of the margin between the apex lobe and the next nearest lobe. Mini leaflets, defined as small leaflets along the midvein between lobes, were assessed as present or absent, and the shape of leaflets was assessed as lobed or non-lobed (Fig. 2). All measurements were quantified using ImageJ software (Schneider *et al.* 2012).

#### Resource allocation measurements

To evaluate genetic differentiation for traits associated with resource allocation we assessed specific leaf area, chlorophyll content obtained through fluorescence emissions (Gitelson *et al.* 1999), leaf dry matter content and water-use efficiency for a subset of individuals during the summer of 2018. These traits encompass a physiological trade-off between efficient resource acquisition, specifically carbon sequestration while minimizing the consequences of water loss via transpiration (Reich *et al.* 1997; Messier *et al.* 2010). One to five individuals per population were evaluated. Due to the unbalanced number of populations per region, between 9 and 56 individual measurements were taken per trait per region **[see****Supporting Information—Table S1****]**.

Specific leaf area (SLA), calculated as a ratio of leaf area to dry mass, was measured over 1 day using a LI-3000C (Li-Cor Biosciences) portable area sensor for 99 individuals (~5 individuals per population for a total of 33 PRA, 56 GLA and 10 MBA individuals). The surface area of one randomly selected mature leaf was estimated alongside fresh and dry mass. Leaves were dried for 68 h at 50 °C, following which dry mass was taken. Specific leaf area is calculated as dry mass per unit leaf area. Leaf dry matter content (LDMC) was also calculated with these data, as LDMC is calculated as the ratio of fresh to dry mass.

Chlorophyll content was quantified over a single 90-min period in the field common garden using a CCM-300 (Opti-Sciences) on 98 individuals, ~5 individuals per population for a total of 33 PRA, 56 GLA and 9 MBA individuals. The CCM-300 records emission ratios of 700 and 735 nm (red and far red wavelengths), which is linearly correlated to chlorophyll content (Gitelson *et al.* 1999). Thus, chlorophyll content values obtained reflect observed wavelength ratios related to chlorophyll content in mg m^−2^.

To quantify integrated water-use efficiency (WUE), we used carbon isotope composition as measured by δ ^13^C (Farquhar 1989). Leaf samples from ~5 individuals per population (53 GLA, 9 MBA and 31 PRA individuals) were sampled from the field common garden and oven-dried at 55 °C over a 24-h period. Following this, leaf samples were homogenized into a fine powder using a TissueLyser II (Qiagen, Hilden, Germany) and 4–5 mg of each sample were weighed and placed into a tin capsule (Costech, Valencia, CA, USA) for ^13^C isotope analysis using a continuous flow isotope ratio mass spectrometer (Sercon Ltd, Cheshire, UK) at UC Davis Stable Isotope Facility (Davis, CA, USA). To assess the repeatability of isotope measurements across samples, 23 technical replicates were evaluated. A correlation of *r* = 0.793 across technical replicates provides confidence in the repeatability and precision of the assay. Reported δ ^13^C values are expressed as relative to the Vienna Pee Dee Belemnite.

#### Stomatal measurements

Stomatal density and size were measured for adaxial and abaxial leaf surface impressions for individuals within the common garden experiment. Newskin ‘liquid bandage’ was applied to either adaxial or abaxial surfaces for two leaves per individual within the common garden for a total of 650 leaf surface impressions (417 GLA, 91 MBA and 142 PRA individuals, **see****Supporting Information—Table S1**). Surface impressions were mounted onto slides and photographed using a Zeiss Stereo Discovery (V8) digital microscope with a Canon Rebel T3 E0S 1100D digital camera. Stomatal density was calculated as the number of stomata from either the abaxial or adaxial surface divided by the area of the impression image (0.32 × 0.42 mm). Guard cell length was measured using ImageJ software (Schneider *et al.* 2012) and calculated based on an average of three stomata per surface impression. Stomatal area index (SAI), which is the total amount of area covered by stomata on a leaf surface, was calculated as the product of guard cell length and stomatal density (Bertel *et al.* 2017).

Stomatal conductance was measured using a Decagon SC-100 Porometer (METER Group) between 09:00 a.m. and 11:30 a.m. over the course of 5 days (8 August 2018 to 12 August 2018) for 99 individuals in the common garden experiment (~5 individuals per population for a total of 33 PRA, 56 GLA and 10 MBA individuals, **see****Supporting Information—Table S1**). Stomatal conductance provides an estimate of the balance between plant CO_2_ uptake and water loss, calculated as the amount of water vapour (H_2_O) exiting through the stomata of a defined leaf section over a 30-s period. Five individuals were sampled per population **[see****Supporting Information—Table S1****]**, with one individual within each population measured per day to minimize effects of temporal environmental variation on population-level estimates. A subset of 10 individuals were measured daily over the course of the experiment. The aim of the repeated measures was to account for possible influence of temporal variation in temperature and humidity on stomatal conductance. We used a linear model to assess repeatability, with individual as a fixed effect, and temperature and humidity as random effects. Temperature and humidity had little effect on stomatal conductance measurements (*R*^2^ = 0.151, and *R*^2^ = 0.000, respectively), and repeatability within individuals was fairly low (*R*^2^ = 0.372).

### Statistical analysis

#### Assessing the scale of genetic differentiation across complex landscapes: regional and population effects

In a common garden experiment, trait differences observed can be associated with genetic differences as the shared environment controls much of the environmental variation that might otherwise contribute to the expression of trait differences (De Kort *et al.* 2014). Phenotypic traits were assessed for normality and homogeneity of variance using the Shapiro–Wilk test and Bartlett test, respectively. Of the 15 traits evaluated, specific leaf area (SLA) was log-transformed to meet assumptions of normality. In addition, a square root transformation was used for stomatal density, stomatal conductance, adaxial and abaxial stomatal area indices (SAI) and sinus depth. Midvein length failed both the Shapiro–Wilk test and Bartlett test, but was visually assessed as normal.

To estimate the proportion of variance explained by region and population, we fit a linear mixed effect model using the lme4 package in R (Bates *et al.* 2015; R Core Team 2018) for each trait. The full model for each trait was:

$$\begin{array}{l} y_{ijk}=R_{i}+p_{j}+e_{ijk} \end{array}$$

where *y*_*ijk*_ is the predicted trait value for individual from region *i* and population *j*; with *R* being the effect of region *i*, *p* is the effect of population *j* and *e* as the residual variance for individual *k*. Within the mixed model, region (*R*_*i*_: PRA, GLA or MBA) was classified as a fixed effect and population as a random effect, as populations represent a random selection of the number of populations (*p*_*j*_) within each region. Interaction terms were not included in the model because no population occurred in more than one region. Normality of residuals was visually assessed for all traits. All statistical tests were conducted in R (R Core Team 2018).

To determine the impact of ecogeographic scale on trait differentiation, we estimated the proportion of variance explained by fixed (region) or random (population, residual) effects for each trait using the rptGaussian function in the rptR package (Stoffel *et al.* 2017; R Core Team 2018). The rptR package estimates the proportion of variance explained by a given effect, which can be considered equivalent to a goodness of fit, or *R*^2^ (Nakagawa and Schielzeth 2013). We estimated the *R*^2^ value using region as a fixed effect. As estimates of regional difference could have an impact on random (population) effects, regional differences were accounted for by including fixed effect estimates in the denominator of the *R*^2^ (i.e. marginal *R*^2^s; Nakagawa and Schielzeth 2013), or unadjusted repeatabilities (Nakagawa and Schielzeth 2010) for population effects. These estimates of *R*^2^ can be biologically interpreted as the proportion of variation in traits that is attributed to genetic differentiation at the corresponding ecogeographic scale (region or population), with residual variation representing differences in trait values from effects not evaluated in this study.

#### Comparing the proportion of variance explained by region and population on quantitative traits and across trait classes

To determine the significance of fixed (region) effect estimates, an analysis of variance (ANOVA) was conducted on the linear mixed effect model (Table 2). The proportions of variance were then bootstrapped (*n* = 1000) to provide a 95 % confidence interval for variance explained for each trait dependent on the predictor variable. *P*-values for random effects (population) were obtained from likelihood-ratio tests.

**Table 2.** Proportion of variance explained by each predictor (region, population or residual) per each trait model. Quantitative trait category represents which trait class the trait is categorized as. Effect sizes with significant *P*-values < 0.05 are represented in italics with two asterisks after for region and population.

Trait	Physiological trait category	Effect size of region	Effect size of population	Residual variance
Leaf sinus depth (Sinus Depth)	Morphological	0.013	0.079**	0.908
Midvein length (MVL)	Morphological	0.056**	0.1**	0.844
Presence of mini leaflets (MLP)	Morphological	0.008	0.047**	0.944
Presence of lobed mini leaflets (MLL)	Morphological	0.008	0.161**	0.831
Chlorophyll fluorescence (CC)	Resource allocation	0.173**	0.177**	0.649
Specific leaf area (SLA)	Resource allocation	0.014	0.292**	0.693
Leaf dry matter content (LDMC)	Resource allocation	0.138**	0.117	0.745
Carbon isotope discrimination (dC13)	Resource allocation	0.201**	0.166**	0.633
Stomatal conductance (Cond)	Stomatal	0.026	0.000	0.974
Stomata density (abaxial) (SD-B)	Stomatal	0.122**	0.15**	0.728
Stomata density (adaxial) (SD-T)	Stomatal	0.125**	0.151**	0.724
Stomata size (abaxial) (GCL-B)	Stomatal	0.072**	0.096**	0.832
Stomata size (adaxial) (GCL-T)	Stomatal	0.108**	0.174**	0.718
Stomata area index (abaxial) (SAI-B)	Stomatal	0.118**	0.134**	0.749
Stomata area index (adaxial) (SAI-T)	Stomatal	0.094**	0.129**	0.777

To assess differences in trait classes at different scales, proportions of variance for each trait were averaged within each trait class. Due to a significant difference in homogeneity of variance across trait classes, Kruskal–Wallis tests were used to compare trait class variance attributed to region and population. Quantitative traits were grouped into ‘morphological’, ‘resource allocation’ or ‘stomatal’ trait classes based on primary function within the leaf **[see****Supporting Information—Table S1****]**. A Dunn test *post hoc* evaluation was conducted using the Dunn.test (Dinno 2014) package to compare variance explained across trait classes. All tests were run using R (R Core Team 2018, version 3.5.0).

#### Assessing the role of climate in landscape-level trait heterogeneity

The geographic provenance of each population was used to model average annual climate variables **[see****Supporting Information—Table S2****]**. Latitude, longitude and elevation were input into the program ClimateNA (Wang *et al.* 2016) to summarize variation among GLA, MBA and PRA regions and a principal components analysis was performed to provide unconstrained axes of climatic differentiation. Additionally, we carried out a redundancy analysis (RDA; Legendre and Gallagher 2001) to study the relationship between quantitative trait variation, climatic variability and ecogeographic regions. The RDA provides the opportunity to compare variation in response variable (traits) when constrained by explanatory variables (climate). For these analyses, we used population averages for quantitative traits, excluding those populations that had missing data for select traits (AB-HSC, AB-RL, AB-RO, NAP-ASS) and standardized traits for comparability. We used the ‘MASS’ package to conduct the RDA ordinations.

## Results

### Quantitative traits differentiation across complex landscapes: regional and population effects

**Figure 3.**
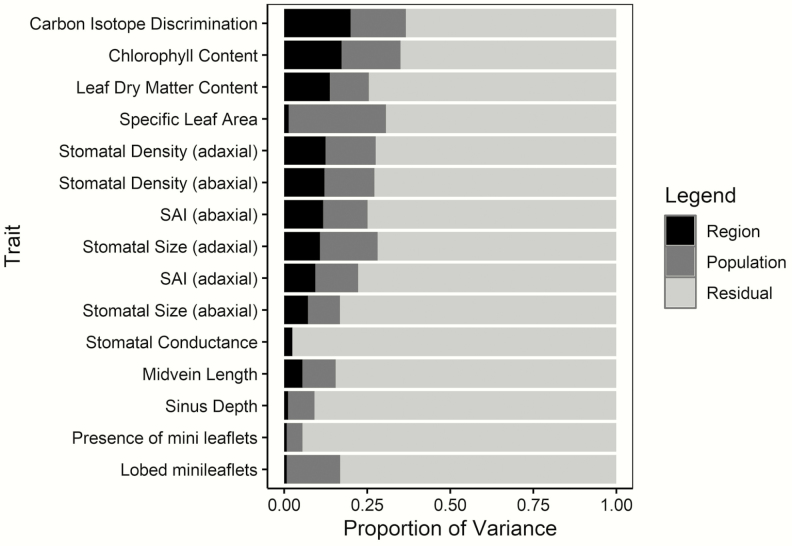
Proportion of variance explained by region (black), population (dark grey) and residual individual (light grey) variance for quantitative traits measured in a common garden experiment. Linear mixed models were used to calculate the proportion of variance explained with region as a fixed effect and population as a random effect, with all other variation considered individual residual variance.

The proportion of phenotypic variance explained by region varied between *R*^2^ = 0.013 to 0.201 for all traits (Table 2; Fig. 3), and population from 0 to 0.292 (Table 2). Ten of the 15 traits measured had a significant proportion of variance explained at the regional scale (Table 2). In addition, 13 of the 15 traits had a significant proportion of variance explained at the population scale (Table 2). This indicates that region and population differences explain a substantial proportion of variation for some traits, but not all. Below we elaborate on the variation explained by region and population organized by trait class.

### Morphological traits

The proportion of variance for midvein length explained by region was significant (*R*^2^ = 0.056, *P* = 0.020). However, regional differences did not have a significant effect on variability observed for other morphological traits, including leaf sinus depth (*R*^2^ = 0.013, *P* = 0.245), presence of mini leaflets (*R*^2^ = 0.009) and the presence of lobed or unlobed leaflets (*R*^2^ = 0.008) (Table 2; Fig. 3). In contrast, the proportion of variance explained by population-level differences for sinus depth and midvein length was low, but significant (*R*^2^ = 0.079, *P* < 0.001 and *R*^2^ = 0.100, *P* < 0.001, respectively) (Table 2). In addition, population-scale effects explained a significant proportion of variation in presence of mini leaflets and whether mini leaflets were lobed or not *R*^2^ = 0.047 (*P* < 0.001) and *R*^2^ = 0.161 (*P* < 0.001), respectively.

### Resource allocation traits

The proportion of variance explained for specific leaf area (SLA) by region was negligible and insignificant (*R*^2^ = 0.014, *P* = 0.718). This contrasts with carbon isotope discrimination where we observed a significant proportion of variability explained by regional differences (*R*^2^ = 0.201, *P* = 0.028). Other resource allocation traits exhibited a similar pattern, including leaf dry matter content and chlorophyll content (*R*^2^ = 0.138, *P* = 0.017 and *R*^2^ = 0.173, *P* = 0.012, respectively) (Table 2; Fig. 3).

While regional effects explained a significant proportion of trait variability for resource allocation traits, population-scale effects also contributed substantially to trait variances (Table 2). Interestingly, the proportion of variability explained by population-level variation for specific leaf area was significant (*R*^2^ = 0.292, *P* = 0.001). Region and population explained the same proportion of variance for chlorophyll content (region and population *R*^2^ = 0.177, Table 2) and a similar proportion of variability was explained by both region and population for leaf dry matter content (Table 2, region *R*^2^ = 0.138; population *R*^2^ = 0.117). Region explained a greater proportion of variation in carbon isotope discrimination relative to population-level differences, though both were significant (region *R*^2^ = 0.201, *P* = 0.007; population *R*^2^ = 0.166, *P* = 0.028, Table 2).

### Stomatal characteristics

A significant proportion of trait variation was explained by region for stomatal density and SAI (density: *R*^2^ = 0.122, *P* = 0.006 abaxial; *R*^2^ = 0.125, *P* < 0.001 adaxial; SAI: *R*^2^ = 0.118, *P* = 0.006 abaxial; *R*^2^ = 0.094, *P* = 0.011 adaxial; Table 2; Fig. 3). However, the proportion of variance explained by region for stomatal size differed substantially between leaf surfaces (*R*^2^ = 0.072, *P* = 0.016 abaxial; *R*^2^ = 0.108, *P* = 0.015 adaxial; Table 2). Finally, while a significant proportion of variability was explained by region across all stomatal traits, population-level differences were also significant (Table 2). This excludes stomatal conductance, where variability was not explained by either regional- or population-level differences (*R*^2^ = 0.026, *P* = 0.247; *R*^2^ < 0.001, *P* = 0.500).

### Comparison across trait classes

**Figure 4.**
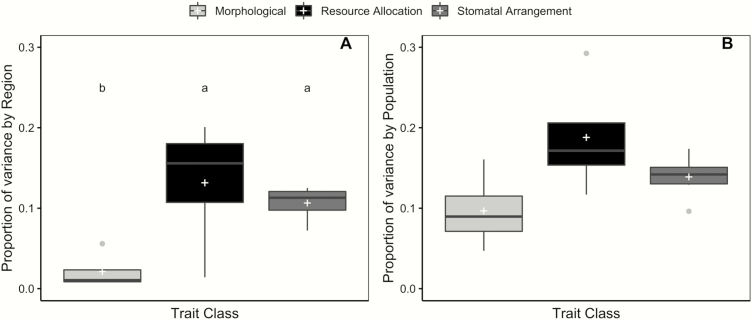
Proportion of variance explained across functional trait classes for regional (A) and population (B) effects. Significant differences were observed across trait classes, including morphological (light grey), resource allocation (black) and stomatal traits (medium grey) for regional effects, whereas no significant differences were observed for population effects. Statistical differences are denoted by a letter change (*P* < 0.05) and same letters indicate no significant difference. White crosses indicate mean variance explained for each trait class.

We compared the proportion of trait variances explained by regional- and population-scale differences for traits grouped into three distinct trait classes: morphological, resource allocation and stomatal trait classes. Stomatal conductance was removed from this comparison as no variation was explained by regional or population scales and the trait exhibited limited repeatability. From the Kruskal–Wallis test, the proportion of variance explained by region significantly varied across trait classes (χ ^2^ = 7.914, *P* = 0.020). Resource allocation and stomatal trait classes explained a significantly greater proportion of regional trait variance relative to morphological traits (Fig. 4). Resource allocation traits exhibited the greatest proportion of regional trait variance, with the stomatal trait class only slightly lower. Despite regional differences in the proportion of variance explained across trait classes, there was no significant difference in the proportion of phenotypic variance explained across trait classes at the population scale (χ ^2^ = 4.829, *P* = 0.089, Fig. 4). This reflects the increased variability around the mean at the population scale.

### Relationship between quantitative trait differences and climate variability

**Figure 5.**
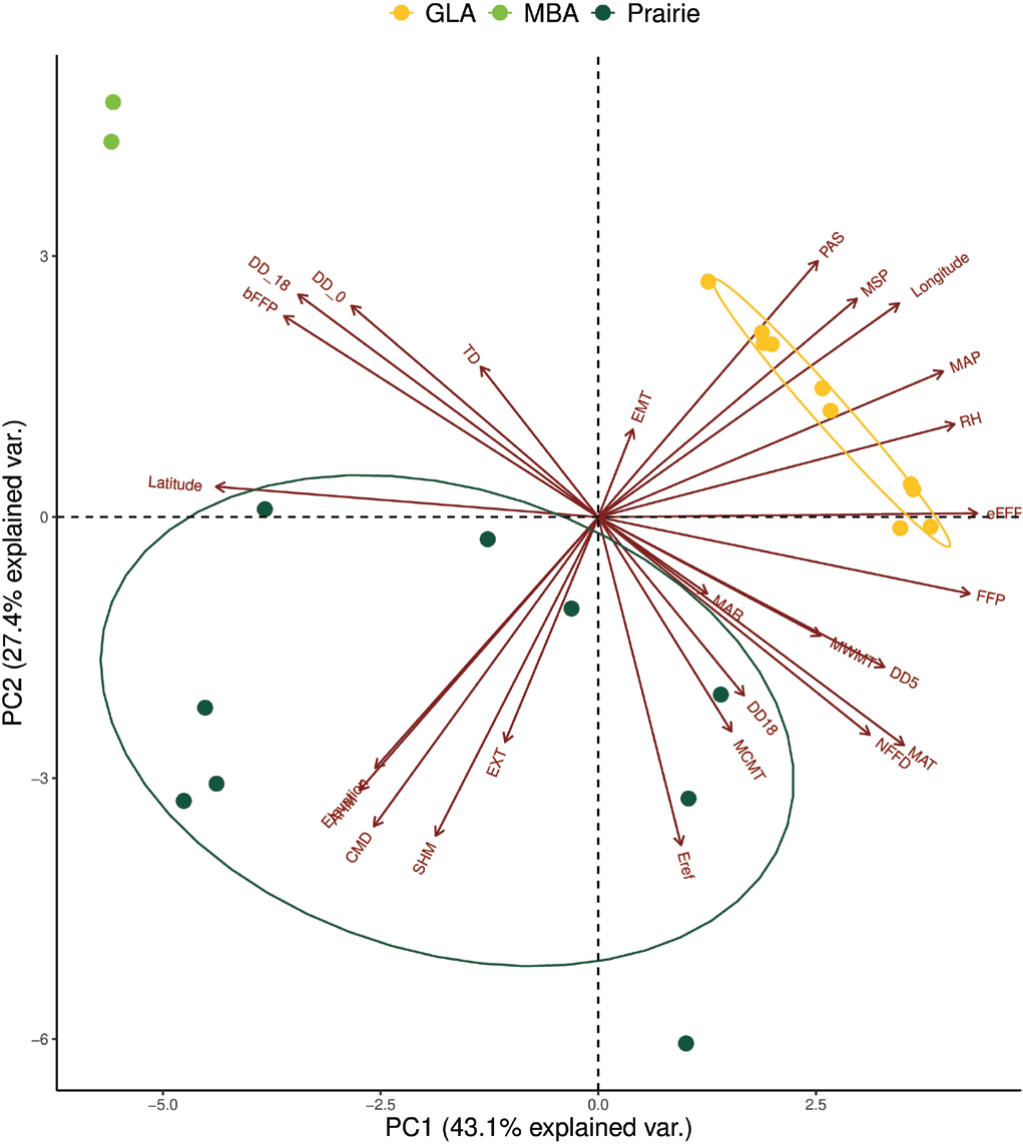
Principal components analysis of climatic data modelled from population origin in ClimateNA summarizing differentiation across populations sampled from three ecogeographic regions: Great Lake Alvar (GLA), Manitoba Alvar (MBA) and Prairie (PRA) regions. The first principal component (PC1) summarizes 43.1 % of variance in climate across populations and second principal component (PC2) 27.4 % of the variance. Orange circles and associated ellipses represent GLA, light green MBA, and dark green PRA populations. Arrows associated with climatic variables indicate loadings associated with each climatic variable on PC1 or PC2 and are fully described in **Supporting Information—Table S2**.

**Figure 6.**
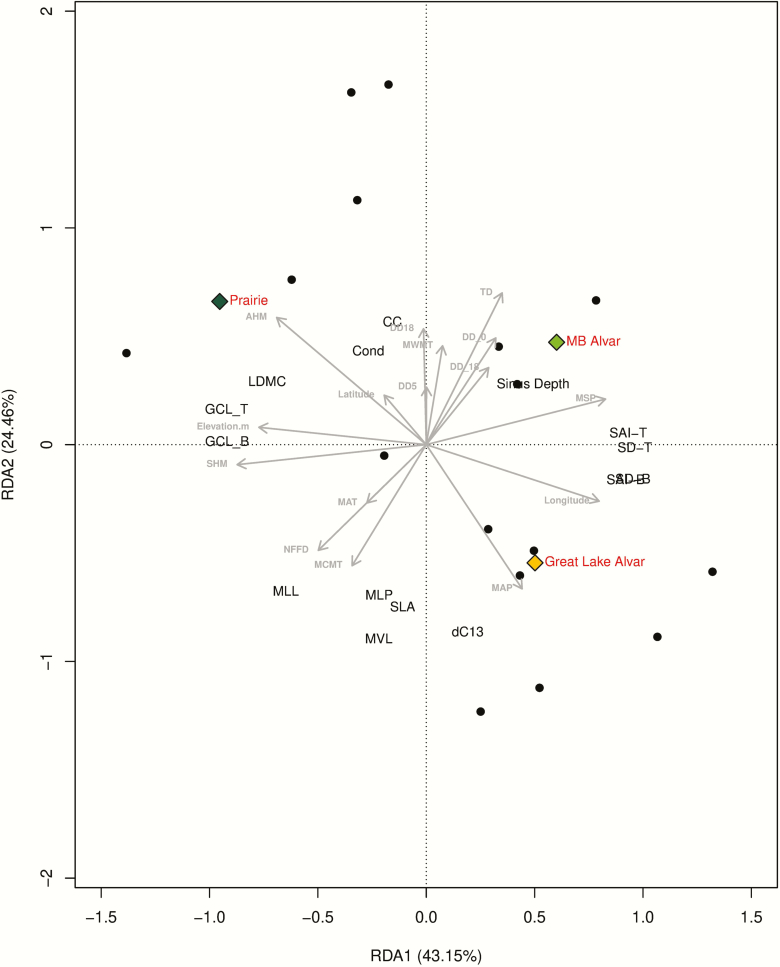
An ordination triplot of redundancy analysis (RDA) for quantitative trait variation explained within a constrained climatic space. Quantitative traits are population averages including carbon isotope composition (dC13), specific leaf area (SLA), leaf dry matter content (LDMC), chlorophyll content (CC), stomatal conductance (Cond), guard cell length on the top and bottom of the leaf (GCL-T, GCL-B), stomatal density on the top and bottom of the leaf (SD-T, SD-B), stomatal area index on the top and bottom of the leaf (SAI-T, SAI-B), sinus depth (Sinus Depth), midvein length (MVL), mini leaflets present (MLP) and mini leaflets lobed (MLL). Black circles represent populations and coloured diamonds represent the three ecogeographic regions. Grey arrows are associated with climatic variables, indicating loadings associated with each climatic variable on RDA1 or RDA2 and are fully described in **Supporting Information—Table S2**.

Population climate averages were used to summarize region- and population-specific differences in climate as a potential factor contributing to trait differences. GLA, MBA and PRA habitats were clearly differentiated based on climate, with 43.1 and 27.4 % of the variation among regions and populations explained by the first and second principal component (PC) axes, respectively (Fig. 5). Substantial loading of climatic variables associated with water availability distinguished GLA and PRA habitats, including mean summer precipitation (MSP), mean annual precipitation (MAP), Hargreaves climatic moisture deficit (CMD) and annual/summer heat measure indices (Fig. 5). This contrasts with the Manitoba Alvar habitat, which was differentiated based primarily on temperature (MAT) and day length (DD_0). Given the important role that climate may play influencing trait differentiation across regions, we used an RDA to assess the ability of climatic data to explain quantitative trait variation across populations within regions. Substantial variation in traits was explained when constrained by climatic variation indicated by the cumulative variation explained by the first two RDA axes (RDA1 = 43.15 %, RDA2 = 24.46 %, Fig. 6). Indeed, the first RDA axis provided evidence that the moisture gradient that differentiates GLA and PRA regions, indicated by mean annual precipitation (MAP) and annual heat moisture indices (AHM), contributed to differences in stomatal density (SD-T, SD-B) and leaf dry matter content (LDMC) and chlorophyll content (CC). The RDA indicates that climate is likely a major determinant of trait variation primarily reflecting a gradient in water availability. Thus, trait variation reflects trade-offs in resource allocation, with increased stomatal density in GLA relative to PRA, and greater leaf dry matter content and chlorophyll content in PRA relative to GLA (K. Volk *et al.*, in preparation).

## Discussion

Understanding the scale over which trait differences and functional trait classes evolve will impact development of seed transfer guidelines for restoration. We examined quantitative trait differentiation in a common garden experiment for individuals sourced from much of the range of *G. triflorum*. We found phenotypic variance was explained by regional- and population-scale differences. Consistent with previous literature, genetic variation is partitioned at broad regional and fine population scales across complex landscapes (Messier *et al.* 2010; Hovick *et al.* 2018; Baughman *et al.* 2019). While genetic differences in traits important to adaptation have evolved between ecologically distinct regions (Etterson 2004; Durka *et al.* 2017), fine-scale evolutionary processes likely also contribute to the distribution of trait variation (Messier *et al.* 2010; Hovick *et al.* 2018). Our results suggest that the distribution of trait variation differed by functional trait class at the regional level. Stomatal trait and resource allocation trait classes had greater variance explained by regional differentiation relative to morphological traits. Differences in stomatal and resource allocation traits across regions may reflect directional selection associated with climatic adaptation over varying ecogeographic scales. Thus, selection across ecogeographic scales likely contributes to the distribution of trait variability in complex landscapes.

### Trait differentiation across scales in complex landscapes

The distribution of trait variances suggests that landscape-level processes likely play an important role in shaping quantitative trait differences at the regional scale (Manel *et al.* 2003; De Kort *et al.* 2014; Maia *et al.* 2017). Across 15 quantitative traits observed in the common garden, region explained between nearly 0 and 20 % of the observed variation (Table 2). In this system, contrasting environmental extremes of alvar and prairie habitats likely contribute to the evolution of regional genetic differences (Anderson 2006; Hamilton and Eckert 2007). Indeed, provenance climate averages suggest that moisture availability largely structures differentiation between the alvar and prairie regions. Given this, selection associated with regional differentiation in water availability has likely influenced the distribution of trait variation.

While a substantial proportion of quantitative trait variation was explained by regional differences, equal or greater variance was explained by population-scale effects. This suggests that alvar and prairie regions are not only differentiated from each other, but populations within regions are also highly differentiated from each other. While the direction and magnitude of selection likely vary across regions, site-specific selection across populations can affect trait differentiation. Furthermore, gene flow and drift may influence the distribution of genetic variation (Manel *et al.* 2003). Stochastic changes associated with reduced connectivity and demography can contribute to differences observed. Previous research from Hamilton and Eckert (2007) indicated that within the same geographic distance, alvar populations were more genetically different from each other at neutral genetic loci than prairie populations. They attributed population-scale differentiation within regions to the combined influence of reduced gene flow and fine-scale environmental selection. For geographically disjunct alvar populations the combined influence of reduced gene flow and selection may lead to greater variance explained for the population-scale relative to more continuous prairie environments.

### Variance across trait classes for regional- and population-level differences

To broadly compare the proportion of variance explained by region and population for functional trait classes, we grouped 15 individual quantitative traits into resource allocation, stomatal and morphological trait classes. Our results suggest that differences have evolved across functional trait classes, but that those differences do not manifest equally across all trait classes. Resource allocation and stomatal trait classes exhibited significantly greater regional differentiation relative to the morphological trait class (Fig. 4). Resource allocation and stomatal traits are likely important to adaptation and may be under strong divergent selection across regional environments. Carbon isotope discrimination is typically viewed as a proxy measure of water use efficiency (Farquhar *et al.* 1989) and differentiation in this ratio suggests genetic differences have likely evolved as an adaptive response to extremes in water availability in the alvar ecosystem. Resource allocation traits measured here represent part of the ‘leaf economic spectrum’ (Grime *et al.* 1997; Wright *et al.* 2004), where trade-offs exist between resource investment and leaf lifespan. We expect that differential investment in leaf traits associated with alvar and prairie environments likely contributes to substantial regional differences for this trait class.

In addition to resource allocation, traits related to stomatal characteristics, specifically size and number of stomata may be under differential selection at the regional scale. Alvar plants experience seasonal flood and drought cycles, which likely select for increased efficiency in managing extremely variable water availability. The most efficient stomatal arrangement for rapid response to environmental change is more stomata of smaller size (Drake *et al.* 2013; Carlson *et al.* 2016). Therefore, we expect divergence in the direction of selection for stomatal traits between alvar and prairie habitats. Consistent with these predictions, alvar populations exhibit smaller size and greater number of stomata, while prairie populations exhibit larger, but fewer stomata (K. Volk *et al.*, in preparation). The large amount of variation explained by region for these traits appears to be driven by environmental contrasts. Furthermore, redundancy analysis suggests that prairie populations may invest more in resource allocation traits, with greater accumulation of leaf dry matter content and chlorophyll content, while alvar populations invest in finer-scale water control via stomatal traits.

Little quantitative trait variance was explained by regional effects within the morphological trait class. These data suggest that morphological traits, while important to plant form and function, are not under strong diversifying selection across regional or population scales for *G. triflorum* or do not exhibit enough genetic variation for which natural selection to act upon. This contrasts with the stomatal characteristic and resource allocation trait classes, suggesting that differentiation within morphological traits may more adequately reflect differentiation due to stochastic processes. Differentiation in morphological traits at the regional scale may be a product of drift or demographic processes, particularly if differences in these traits do not affect relative fitness of individuals (Hereford 2009).

Local adaptation can occur on multiple scales (McKay *et al.* 2005), and genetic differences can evolve between populations as a result of fine-scale response to selection. While a greater proportion of variance is explained at the population scale, no significant differences were observed in that proportion across trait classes (Fig. 4). While populations have evolved differences, the evolutionary processes contributing at the population scale likely represent a combination of stochastic and deterministic processes. Reduced connectivity among alvar populations and prairie fragments, alongside site-specific selection not directly studied here could have substantial influence on the distribution of population genetic variation for quantitative traits (Hamilton and Eckert 2007).

### Evolutionary factors impacting trait variance across scales

Our data support the evolution of genetic differences across heterogeneous landscapes in response to varying selective pressures, but we cannot rule out the possibility that other evolutionary processes or shifts in reproductive biology have shaped the distribution of quantitative traits. If reproductive biology, presumed to be a combination of selfing and outcrossing in G. triflorum, exhibits variation across regions or populations, then the signature of differentiation would likely be exacerbated by mating system (Hamilton et al. 2007). Furthermore, alvar habitats were likely colonized by *G. triflorum* during a range expansion from prairie environments through the warming Hypsithermal period (Hamilton and Eckert 2007). Stochastic demographic processes during colonization, including founder events and population bottlenecks, likely contributed to observed differentiation in contemporary quantitative traits. In addition, the alvar populations of *G. triflorum* are disjunct not only from prairie environments, but also from each other. If effective population size is small, and barriers to gene flow exist between regions, genetic drift may contribute to accumulation of genetic differences (Lande 1992; Knapp and Rice 1996; Young *et al.* 1996). The impact of drift is particularly relevant for those populations at the margin of the species’ range, including alvar and western prairie populations, where differential survival or reproduction may lead to differentiation among populations within regions (Lesica and Allendorf 1995; Jump *et al.* 2003).

Our findings support regional- and population-scale differentiation for traits important to adaptation across the range of *G. triflorum*; however, the relationship to fitness, including reproductive success or number of reproductive events undertaken, for the individual plants studied has not been estimated. The traits examined here have frequently been related to fitness through life history trade-offs and provisioning resources to reproduction (Dudley 1996; Wright *et al.* 2004; Martin *et al.* 2007; Muir 2015), but do not directly capture reproductive life history variation in *G. triflorum.* As *G. triflorum* is a perennial species, quantifying lifetime fitness would provide a means to relate trait variation to fitness consequences in a given environment (Bucharova *et al.* 2017b; Yoko 2019). Additionally, to truly test whether populations are locally adapted, reciprocal transplant experiments would be necessary (Griffith and Watson 2005; Ackerly *et al.* 2006; Hamilton and Eckert 2007).

## Conclusions

Sourcing seeds for restoration frequently aims to identify seed with the appropriate genetic variation for the restoration environment (Lesica and Allendorf 1999; Gallagher and Wagenius 2016). Understanding how genetic variance for quantitative traits is distributed will aid in establishing guidelines for seed transfer during restoration. If seed transfer is proposed consideration of the impact the scale of trait differences for functional traits may have on success in the restoration environment is necessary (Bucharova *et al.* 2017b). Environmental differences associated with regional climatic variation have clearly influenced the distribution of functional trait variation for *G. triflorum*. These patterns point towards the importance of minimizing environmental differences when transferring seed from origin to restoration site, particularly those differences associated with water availability. Assessing regional climatic differences provides a first step to determining seed transfer recommendations. While regional differences explain a substantial proportion of trait variation, population-level differences are important. Population differentiation within regions provides an important source of variation that may be valuable to developing ‘regional admixed provenances’ that balance local adaptation while maintaining variation necessary for future adaptation (Bucharova *et al.* 2019). Thus, seed transfer within grassland ecosystems may prioritize regional-scale climatic adaptation to ensure contemporary ecosystem function, and admixture among populations within regions to ensure long-term evolutionary potential in a changing climate. Finally, different traits or trait classes may be more or less appropriate for the development of seed transfer guidelines and identifying those traits that are suitable for establishing guidelines may depend on the association with climatic variation.

## Supporting Information

The following additional information is available in the online version of this article—

**Table S1.** Number of *Geum triflorum* individuals assessed for each morphological, resource allocation and stomatal trait within the common garden experiment separated by region.

**Table S2.** Source populations of *Geum triflorum* collected in 2015 separated by region, along with latitude, longitude and elevation (m) of collection sites.

plaa009_suppl_Supplementary_Table_S1

plaa009_suppl_Supplementary_Table_S2

## Data

All data and scripts associated with this manuscript are available on GitHub (https://github.com/zeb-yoko/AoBP-quantitative-trait-differentiation.git).
